# Self-Growth of Centimeter-Scale Single Crystals by Normal Sintering Process in Modified Potassium Sodium Niobate Ceramics

**DOI:** 10.1038/srep17656

**Published:** 2015-12-03

**Authors:** Cheol-Woo Ahn, Ho-Yong Lee, Guifang Han, Shujun Zhang, Si-Young Choi, Jong-Jin Choi, Jong-Woo Kim, Woon-Ha Yoon, Joon-Hwan Choi, Dong-Soo Park, Byung-Dong Hahn, Jungho Ryu

**Affiliations:** 1 Functional Ceramics Department, Powder & Ceramics Division, Korea Institute of Materials Science (KIMS), Changwon, Gyeongnam 641-831, Korea; 2 Department of Materials Science and Engineering, Sunmoon University, Asan, Chungnam 330-708, Korea; 3 School of Mechanical and Aerospace Engineering, Nanyang Technological University, 50 Nanyang Avenue, Singapore 639798; 4 Institute for Superconducting & Electronic Materials, Australian Institute of Innovative Materials, University of Wollongong, NSW 2500, Australia

## Abstract

In this manuscript, an interesting phenomenon is reported. That is the self-growth of single crystals in Pb-free piezoelectric ceramics. These crystals are several centimeters in size. They are grown without any seed addition through a normal sintering process in modified potassium sodium niobate ceramics. It has been achieved by the composition designed to compensate the Na^+^ loss which occurs during the liquid phase sintering. The composition of the crystals is (K_0.4925_Na_0.4925−x_Ba_0.015+x/2_)Nb_0.995+x_O_3_ [x is determined by the Na^+^ loss, due to Na_2_O volatilization]. These crystals have high piezoelectric voltage coefficients (g_33_, 131 *10*^*−3*^*Vm/N*), indicating that they are good candidates for piezoelectric sensors and energy harvesting devices. We hope that this report can offer the opportunity for many researchers to have an interest in these crystals.

Pb-free ceramics have been studied to replace Pb(Zr,Ti)O_3_ (PZT)-based ceramics, because Pb element causes environmental problems^1^,^2^,^3^,^4^,^5^,^6^,^7^,^8^,^9^,^10^,^11^,^12^,^13^,^14^,^15^,^16^,^17^,^18^,^19^. Among the Pb-free ceramics, (K,Na)NbO_3_ (KNN)-based ceramics have attracted the attention of many researchers, because of their high piezoelectric voltage coefficients which are required at the piezoelectric sensors^4^,^5^. Their microstructure as well as crystal structure must be well-studied to obtain a promising piezoelectric performance in KNN-based ceramics. In our previous study, we have described about the sintering behavior of KNN-based ceramics^7^. In KNN-based ceramics, we have observed the abnormal grain growth. The size of grown grains was less than ten micrometers in magnitude. The abnormal grain growth has been attributed to the formation of Na-deficient liquid phases, which are formed due to the Na_2_O volatilization. Since the Na-deficient liquid phase is not a stoichiometric composition, the grain growth can be restricted to a few micrometers in KNN-based ceramics, as shown in Fig. 1. In addition, we have not witnessed the growth of large single crystals through solid-state single crystal growth (SSCG) in KNN-based ceramics, although it is a promising method to obtain large single crystals in PZT-based materials, as shown in Fig. 2(a)^20−22^. This can be also because the Na-deficient liquid phase is not a stoichiometric composition in KNN-based ceramics. Therefore, we have designed a new KNN-based material, in order to compensate the Na^+^ loss which can occur due to Na-deficient liquid phases, during the grain growth in KNN-based ceramics.

In this manuscript, we have described the composition, crystal structure, microstructure, and piezoelectric voltage coefficient (g_33_) of the newly designed KNN-based material. We hope that this report attracts the attention of many researchers as we have synthesized distinctive crystals from KNN-based material. In particular, we expect the quality of these crystals can be improved and scientific approaches can be achieved by a lot of researchers.

## Results

### Composition and Crystal Growth

**Figure 1:**
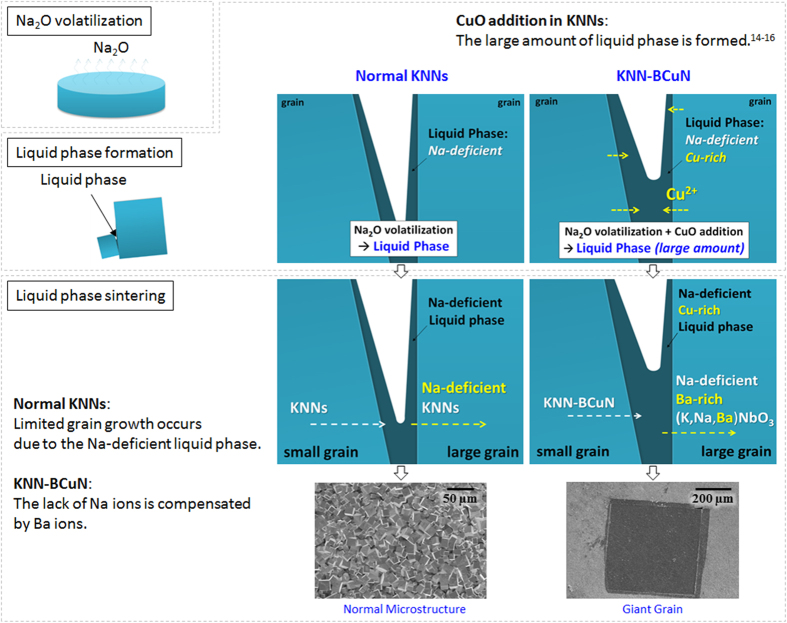
Difference between (K_0.5_Na_0.5_)NbO_3_-based ceramics (KNNs) and 0.985(K_0.5_Na_0.5_)NbO_3_-0.015Ba(Cu_1/3_Nb_2/3_)O_3_ ceramics (KNN-BCuN) in sintering behavior: While the grain growth appears to be limited due to the Na-deficient liquid phase in KNNs, the lack of Na ions is compensated by Ba ions in KNN-BCuN.

**Figure 2:**
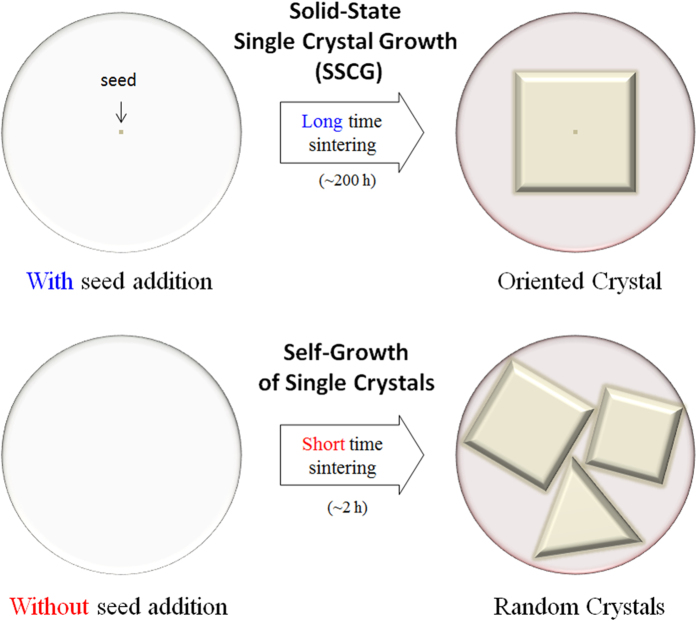
Schematic diagrams of solid-state single crystal growth (SSCG) and self-growth of single crystals: SSCG is a long time sintering technique using a seed specimen. The large single crystal is well-grown by SSCG in Pb(Zr,Ti)O_3_ (PZT)-based ceramics. At the self-growth of single crystals, any seed specimen is not required and the growth is much faster than SSCG. It is found in 0.985(K_0.5_Na_0.5_)NbO_3_-0.015Ba(Cu_1/3_Nb_2/3_)O_3_ [KNN-BCuN] ceramics.

**Figure 3:**
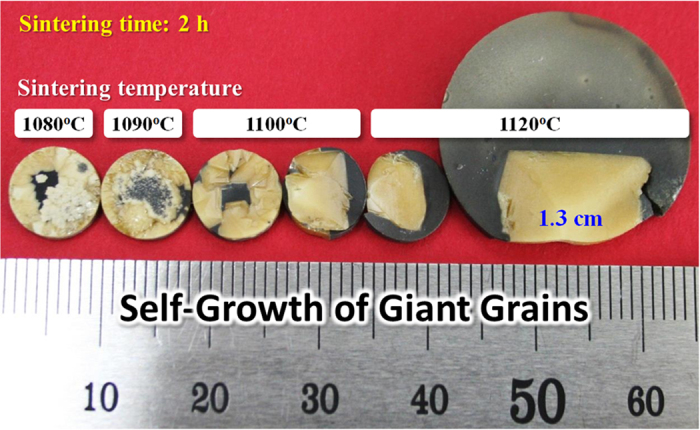
Variation of crystal size with sintering temperature in KNN-BCuN ceramics: The larger crystals are grown at a higher sintering temperature. The sintering time is 2 h.

**Figure 4:**
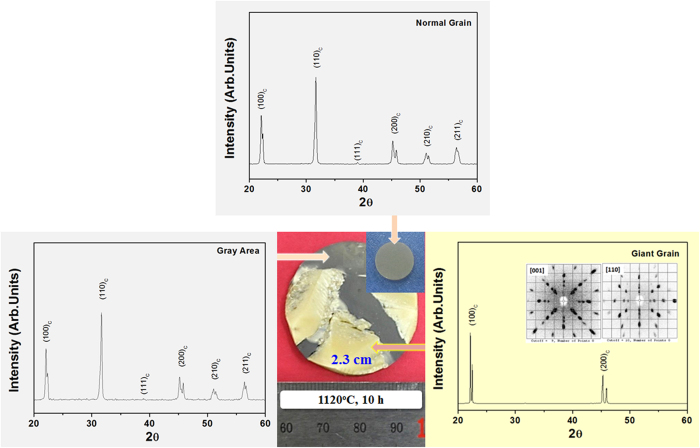
Photograph, X-ray diffraction (XRD) patterns, and Laue images of KNN-BCuN specimen sintered at 1120 ^o^C for 10 h: Yellow regions are single crystals and gray areas are polycrystalline ceramics. The inset of a photograph shows a normal grain specimen in which the growth of giant grains does not occur.

We compensated the loss of Na^+^ loss in (K,Na)NbO_3_ by adding another metal ion. In addition, we added CuO in order to sufficiently form liquid phase. This is because CuO accelerates the formation of liquid phase in KNN-based ceramics^14^,^15^,^16^. In the CuO-added KNN ceramics, the liquid phase was formed at ≥950 ^o^C. In contrast, the liquid phase would form at ≥1030 ^o^C when CuO was not added in KNN-based ceramics. Thus, the B^I^_1/3_B^II^_2/3_ site of A(B^I^_1/3_B^II^_2/3_)O_3_ has been chosen to be Cu_1/3_Nb_2/3_. In addition, the Ba^2+^ ion was selected to be Ba(Cu_1/3_Nb_2/3_)O_3_ [BCuN], which is appropriate for the development of A(B^I^_1/3_B^II^_2/3_)O_3_ structure. Furthermore, during liquid phase sintering, the Ba^2+^ ion of Ba(Cu_1/3_Nb_2/3_)O_3_ was expected to compensate the loss of Na^+^ ion in AB^II^O_3_-A(B^I^_1/3_B^II^_2/3_)O_3_ structure (A: Na, K, Ba, B^I^: Cu, and B^II^: Nb), as seen in Fig. 1. This is because Cu ion melts with the Na-deficient liquid phase. While investigating BCuN–modified KNN ceramics, we have found an amazing phenomenon in 0.985KNN-0.015Ba(Cu_1/3_Nb_2/3_)O_3_ [KNN-BCuN] ceramics. That is the self-growth of giant grains, which are single crystals, as indicated in Figs 2, 3, 4. As shown in Figs 3 and 4, these crystals are several centimeters in size.

In general, SSCG has been used to obtain a large single crystal in PZT-based piezoelectric materials. As shown in Fig. 2(a), it is a long time sintering process (approximately several hundred hours) using a seed specimen and the single crystal shows the excellent piezoelectric performance^20^,^21^,^22^. Compared with the PZT-based crystals, which are grown by SSCG, these self-grown crystals can be obtained without any seed specimen in KNN-based materials. In addition, the sintering process requires several hours for the self-grown crystals in KNN-based materials. It is approximately 100 times faster than SSCG (Figs 2 and 3). As explained above, a single large crystal could not be obtained by SSCG in KNN-based materials till date^8^,^9^. However, using KNN-BCuN ceramics, a large single crystal can be easily grown through SSCG as seen in supplementary information. In addition, the plate-type crystals can be also produced by molten salt synthesis (Fig. S2).

### Quality of Grown Crystal

As shown in Fig. 3, the self-growth of giant grains is easily visible to the naked eye. At the higher temperature, the larger grain is observed; the giant grain of 1.3 cm is formed when the specimen is sintered at 1120 ^o^C for 2 hours, as exhibited at Fig. 3. It is very interesting that the large single crystals are grown in KNN-BCuN ceramics, although the sintering time is just 2 hours (without any distinctive technique).

Figure 4 shows a photograph, X-ray diffraction (XRD) patterns, and Laue images of the specimen which was sintered at 1120 ^o^C for 10 hours. As shown in Fig. 4, the sintered specimen can be divided into two distinct regions such as the yellow and gray areas. The inset of this photograph shows another specimen (“Normal Grain” in the Figure), which does not exhibit the self-growth of giant grains in KNN-BCuN ceramics. In general, the gray color is observed in CuO-added KNN ceramics which represent the normal grain specimen. Thus, we could deduce that a normal poly-crystalline ceramic was formed in the gray area. As expected, the peaks for poly-crystalline ceramics are detected in the XRD patterns of gray areas and normal grain specimens. In contrast, at the XRD pattern and Laue images of Fig. 4, the yellow regions represent the self-growth of single large crystals. The color of giant grains is not gray but yellow, although CuO is added during the preparation of this specimen and the poly-crystalline area is gray in color. In addition, the giant grains are not transparent, although they are single crystals. Hence, their microstructures have been investigated.

**Figure 5:**
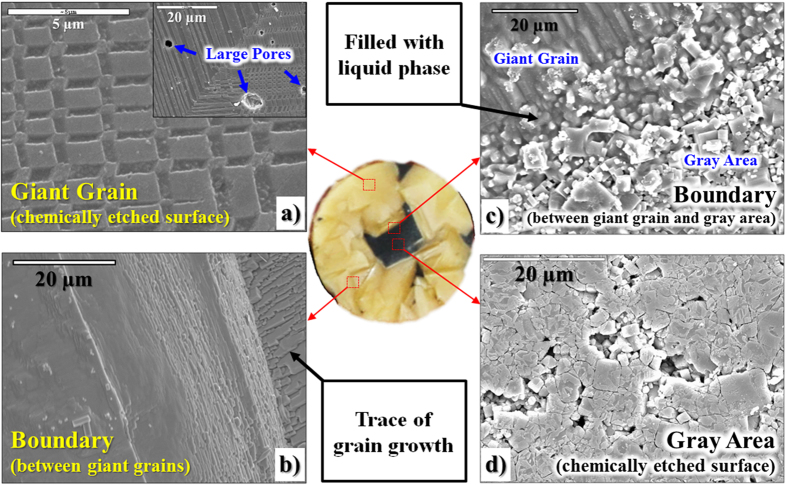
Scanning electron microscope (SEM) images of KNN-BCuN specimen sintered at 1100 ^o^C for 2 h: Large pores are observed in single crystals. The boundary between a single crystal and a gray area is filled with a liquid phase. The trace of grain growth is found at the boundary between single crystals.

**Figure 6:**
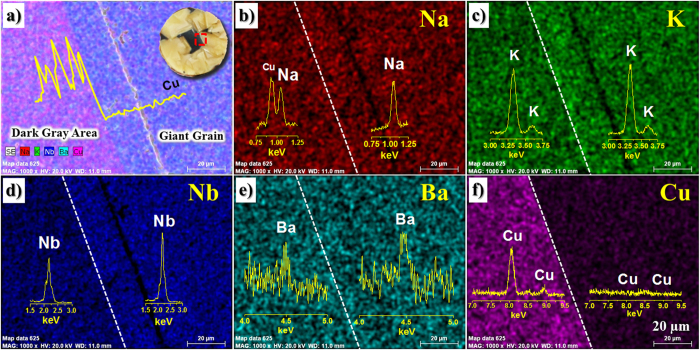
Scanning electron microscope (SEM) image and elements distribution of KNN-BCuN specimen sintered at 1100 ^o^C for 2 h: Cu is not detected and more Ba is observed in the single crystals.

**Figure 7:**
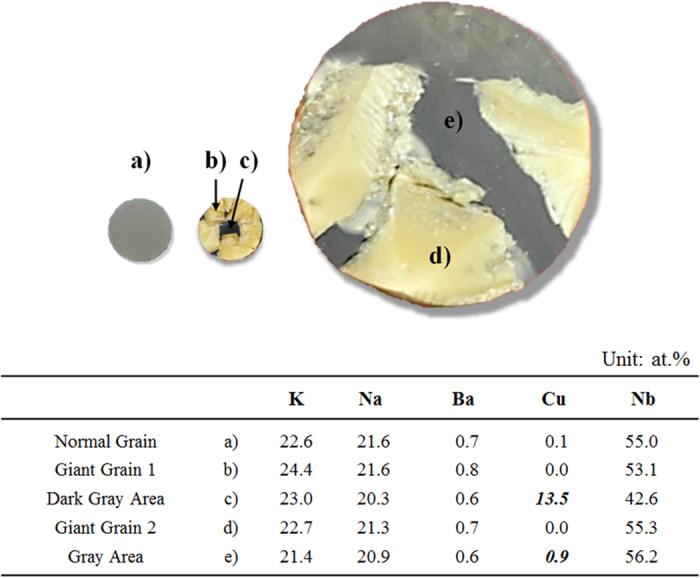
Difference of elements distribution among normal grain, dark gray area, gray area, and giant grain: Less Na is detected at all of the specimens, compared with K. Much more Cu is observed at the gray and dark gray areas than a normal grain specimen as well as a giant grain. The giant grain is a single crystal.

As shown in Fig. 5(a), a giant grain is a single crystal, but it has large pores. Therefore, the giant grains are not transparent. The formation of large pores can be due to the rapid growth of giant grains. In addition, as shown in Figs 6(a–f) and 7, the Cu element is not detected in the giant grains, although it is well-observed at the gray area. In contrast, the Ba element is more detected in giant grains than gray areas, as seen in Fig. 6(e). Therefore, giant grains do not show gray color, because Cu element is not detected in giant grains. In addition, the Ba-modified KNN ceramics have a yellowish appearance in general.

### Compensation of Na Ion Loss

The self-growth of giant grains can be explained by Ostwald ripening which is observed during the sintering process of normal KNN-based ceramics^7^. As shown in Fig. 5(b), the boundary of giant grains is similar to that in KNN-based ceramics. Hence, Ostwald ripening can be also considered in this self-growth of giant grains. In general, in KNN-based ceramics, the liquid phase is well-formed during the sintering process and it is a Na-deficient phase which is formed by the Na_2_O volatilization^7^,^14^,^17^,^18^. In addition, the CuO addition accelerates the formation of liquid phase in KNN-based ceramics. However, this growth of giant grains has not been observed in the other KNN-based ceramics till date.

In general, the grains do not show significant difference at the ratio of elements in KNN-based ceramics. In addition, their perovskite structure is well-maintained after performing the sintering process^7^,^14^,^17^,^18^. Thus, Na_2_O volatilization is enough to form the Na-deficient liquid phase, but it is not so serious to destroy the crystal structure of KNN-based ceramics. Furthermore, since the Na-deficient liquid phase is not a stoichiometric composition, the grain growth can be restricted to micrometer scale in normal KNN-based ceramics.

As shown in Fig. 7, K element is more detected than Na element in KNN-BCuN ceramics. In all the areas of KNN-BCuN ceramics, K element is more commonly found than Na element. Thus, Na^+^ loss is more significant in KNN-BCuN ceramics than in the other KNN-based ceramics. In the CuO-added KNN ceramics, the liquid phase is formed at temperatures above 950 ^o^C, as mentioned above. On the other hand, the sintering temperature of KNN-BCuN ceramics lies in the range of 1070–1125 ^o^C. The temperature range is higher than 950 ^o^C. Hence, compared to other KNN-based ceramics, the loss of Na ion can occur to a much greater extent in these KNN-BCuN specimens, because of the large amount of liquid phase.

As indicated in Figs 6(b,c) and 7, the amount of Na is less than that of K in giant grains. This means the giant grains are continuously grown in a Na-deficient phase during the sintering process. The loss of Na^+^ is permissible because of Cu^2+^ loss and Ba^2+^ input in giant grains. As shown in Figs 6(a,e,f) and 7, Cu is not found in giant grains, but the Ba element is found more frequently in giant grains than in gray areas. Na^+^ loss of A site can be compensated by the Ba^2+^ input of A site and the Cu^2+^ loss of B^I^ site at the AB^II^O_3_-A(B^I^_1/3_B^II^_2/3_)O_3_ structure of giant grains (A: Na, K, Ba, B^I^: Cu, and B^II^: Nb) as shown in Fig. 1. Because of this compensation, the self-growth of giant grains might be possible in KNN-BCuN ceramics. Furthermore, the remaining liquid phase can be recrystallized with the small grains of gray areas during the cooling process. Hence, the gray areas also contain the small amount of Na, as shown in Figs 6(b) and 7. Furthermore, the amount of Cu detected in the dark-gray area of Fig. 7(c) is much more than that observed in the other gray areas. As seen in Fig. 7(c), the dark-gray area appears when the gray area is quite small. This has been attributed to the loss of Cu ion in giant grains during the liquid phase sintering.

Eventually, the chemical formulas of a giant grain and a normal grain might be different. In this case, the chemical formula of the giant grain can be (K_0.4925_Na_0.4925−x_□_x/2_Ba_0.015+x/2_)Nb_0.995+x_O_3_ [x means the Na^+^ loss is either due to evaporation or volatilization.]; the chemical formula of a normal grain can be (K_0.4925_Na_0.4925−x_□_x_Ba_0.015_)(Cu_0.005_Nb_0.995_)O_3_. These chemical formulas might be possible, but further investigations should be carried out to ascertain their definite structures.

### Dielectric and Piezoelectric Properties of Grown Crystal

Since Cu^2+^ loss and Ba^2+^ input occur in the giant grains of KNN-BCuN ceramics, it can be considered that Ba^2+^ ions enter Na^+^ sites of (K,Na)NbO_3_ in giant grains. Thus, as seen in Table 1, metal vacancies must be existed in giant grains and the high dielectric loss is observed because of the metal vacancies. In addition, the pores (which are observed at the giant grains) can be also responsible for the high dielectric loss of giant grains. In general, phase transition temperatures are determined by the proportion of KNN in KNN-based solid solutions^19^. As exhibited at Table 1, compared with pure KNN, these KNN-BCuN ceramics have lower phase transition temperatures. It is interesting that the giant and normal grains did not show the big difference at the phase transition temperatures, although their chemical formulas might be different. It can be also explained by the proportion of KNN. The proportions of KNN are quite similar approximately 0.98 and the transition temperatures are completely matched with our previous results^19^. The normal grain means not the gray area but the KNN-BCuN specimen in which the self-growth of giant grains does not appear, as shown in Figs 4 and 7(a).

The piezoelectric voltage coefficient should be high enough for their use in piezoelectric sensors. As shown in Table 1, this giant grain shows an excellent g_33_ of 131 [*10*^*−3*^ *Vm/N*], which is much higher than that of a PZT based single crystal. Compared with the dielectric and piezoelectric properties of a KNN single crystal which has been prepared by a high temperature flux method^10^, they are quite similar except dielectric loss. The high dielectric loss of a giant grain is due to the metal vacancies. In addition, a high g_33_ is also required at energy harvesting devices because a high d_33_xg_33_ value indicates a high energy density^4^. An excellent d_33_xg_33_ of 26,144 [*10*^*−15*^ *m*^*2*^*/N*] is observed in a giant grain of KNN-BCuN ceramics. Therefore, the giant grain of KNN-BCuN ceramics can be a good candidate for piezoelectric sensors and energy harvesting devices.

## Discussion

In summary, without any seed addition, giant grains which are single crystals are self-grown by the normal sintering process in KNN-BCuN ceramics. The grown single crystal is several centimeters in size. The self-growth of single crystals can be achieved by compensating the Na loss, which is found at the liquid phase of KNN-based ceramics. The Na-deficient liquid phase is formed by Na_2_O volatilization and CuO addition accelerates the formation of liquid phase. In the giant grains, the Ba^2+^ input of A site and the Cu^2+^ loss of B site occur at perovskite structure during the liquid phase sintering and these phenomena are conducive to the self-growth of giant grains in KNN-BCuN ceramics. These self-grown single crystals of KNN-BCuN ceramics have an excellent piezoelectric voltage coefficient. Therefore, the giant grain of KNN-BCuN ceramics can be a good candidate for piezoelectric sensors and energy harvesting devices. However, the self-grown single crystals have higher dielectric loss than a KNN single crystal which has been produced by a high temperature flux method. This high dielectric loss has been attributed to the metal vacancy which is formed due to the Ba^2+^ input and Cu^2+^ loss in these self-grown crystals. In addition, the pores of giant grains must be also considered. We hope that the quality and orientation of these crystals can be improved by other researchers in the near future.

## Methods

The system of 0.985(K_1/2_Na_1/2_)O_3_-0.015Ba(Cu_1/3_Nb_2/3_)O_3_ was synthesized from the oxides of >99% purity by the conventional solid-state route. The powders of K_2_CO_3_, Na_2_CO_3_, Nb_2_O_5_, BaCO_3_, and CuO (all obtained from Sigma Aldrich) were mixed for 6–48 h in a polypropylene jar with zirconia balls. This mixture of powders was dried and calcined at the temperature range of 800–950 ^o^C for 3 h. Calcined powders were milled for 6–48 h, dried and pressed into disks under the pressure of 10 MPa and sintered in the range of 1070~1120 ^o^C for 2–10 h. The giant grain was obtained when the powders were ball-milled for 6–18 h (both 1^st^ and 2^nd^ ball-milling processes). The crystal structure of specimens was examined using Rigaku D/max-RC X-ray diffractometer. The microstructure was observed under a scanning electron microscope (SEM, JSM-5800; JEOL CO., Tokyo, Japan). The samples were poled in silicone oil at 120 ^o^C by applying a dc field of 2–4 kV/mm for 30 min. All the electrical measurements were conducted after aging the samples for 24 h. The piezoelectric and dielectric properties were determined using a piezo d_33_ meter (Micro-Epsilon Channel Product DT-3300) and an impedance analyzer (4294A, Agilent Technologies, Santa Clara, CA, USA).

## Additional Information

**How to cite this article**: Ahn, C.-W. *et al.* Self-Growth of Centimeter-Scale Single Crystals by Normal Sintering Process in Modified Potassium Sodium Niobate Ceramics. *Sci. Rep.* **5**, 17656; doi: 10.1038/srep17656 (2015).

## Supplementary Material

Supplementary Information

## Figures and Tables

**Table 1:** Dielectric properties and piezoelectric voltage coefficients of KNN-BCuN single crystals and ceramics: KNN-BCuN single crystals show high piezoelectric voltage coefficient and dielectric loss, compared with the normal grain specimen.

			T_O–T_ (°C)	T_C_ (°C)	d_33_ (pC/N)	ε_3_T/ε_0_ (at 1 kHz)	tan δ (at 1 kHz) (at 10 kHz)	g_33_ (10^−3^ Vm/N)	d_33_xg_33_ (10^−15^ m^2^/N)
KNN-BCuN	giant grain	[001]	165	396	150	239	0.37 0.14	71	10,633
	normal grain	[110]	170	390	200	173	0.11 0.08	131	26,114
		–			105	315	0.04 0.02	38	3,953
KNN^10^	single crystal*	[001]	205	393	160	240	0.02	75	12,047
	ceramic	–	200	420	80	290	0.02	31	2,493
PMN-PZT^21^	single crystal**		96 (T_R−T_)	215	2,000	6,000	0.005	38	75,296

^*^Prepared by a high temperature flux method.

^**^Prepared by SSCG.
