# Supplementary material for: Self-Growth of Centimeter-Scale Single Crystals by Normal Sintering Process in Modified Potassium Sodium Niobate Ceramics
Source: Sci Rep. 2015 Dec 3;5:17656. doi: 10.1038/srep17656 (PMC4668382; doi:10.1038/srep17656)
Supplement: Supplementary Information [file srep17656-s1.pdf]

Supplementary Information

Self-Growth of Centimeter-Scale Single Crystals by Normal Sintering Process in Modified Potassium Sodium Niobate Ceramics

Cheol-Woo Ahn[1], Ho-Yong Lee[2], Guifang Han[3], Shujun Zhang[4], Si-Young Choi[1], Jong-Jin Choi[1], Jong-Woo Kim[1], Woon-Ha Yoon[1], Joon-Hwan Choi[1], Dong-Soo Park[1], Byung-Dong Hahn[1], and Jungho Ryu[1]*

[1]Functional Ceramics Department, Powder & Ceramics Division, Korea Institute of Materials Science (KIMS), Changwon, Gyeongnam 641-831, Korea
[2]Department of Materials Science and Engineering, Sunmoon University, Asan, Chungnam 330-708, Korea
[3]School of Mechanical and Aerospace Engineering, Nanyang Technological University, 50 Nanyang Avenue, Singapore 639798
[4]Materials Research Institute, Pennsylvania State University, University Park, PA 16802, USA

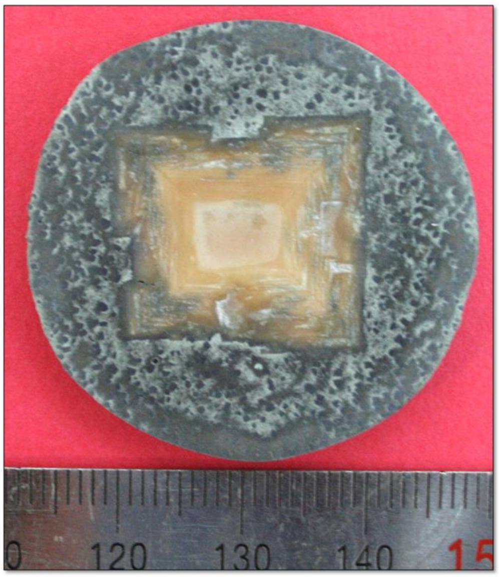

Fig. S1. KNN-BCuN single crystal prepared by solid-state single crystal growth (SSCG): The KNN-BCuN giant grain has been used for the seed. The specimen has been sintered at 1120°C for 100 h.

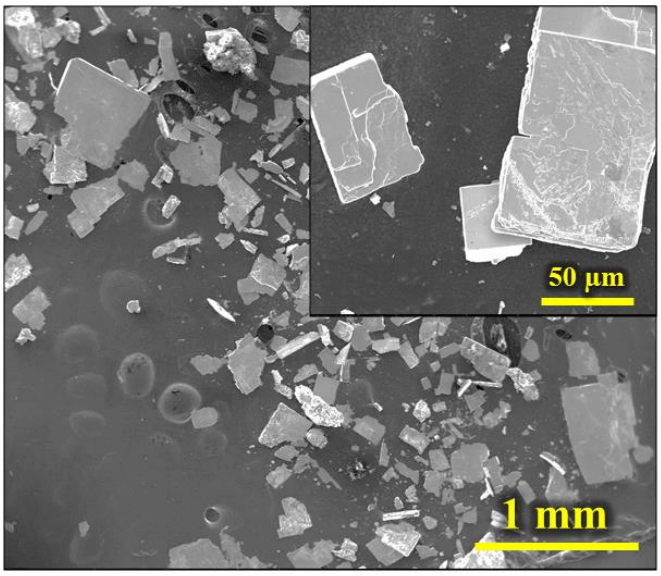

Fig. S2. Plate-type crystals produced by molten salt synthesis (MSS): KF has been used to grow the crystals. Not only $0.985KNN-0.015Ba(Cu_{1/3}Nb_{2/3})O_3$ [KNN-BCuN] but also $0.985KNN-0.015M(Cu_{1/3}Nb_{2/3})O_3$ [KNN-MCuN, M= Sr or Ca] shows the self-growth of giant grains. Using KNN-CCuN, these crystals have been prepared by MSS. The mixture of the calcined KNN-CCuN and KF powders has been used to grow the plate-type crystals of KNN-CCuN (1080°C, 8 h).
